# Good-enough processing, home language proficiency, cognitive skills, and task effects for Korean heritage speakers’ sentence comprehension

**DOI:** 10.3389/fpsyg.2024.1382668

**Published:** 2024-08-01

**Authors:** Gyu-Ho Shin

**Affiliations:** Department of Linguistics, University of Illinois at Chicago, Chicago, IL, United States

**Keywords:** good-enough processing, inhibitory control, working memory, proficiency, heritage speaker, Korean

## Abstract

The present study investigates how heritage speakers conduct good-enough processing at the interface of home-language proficiency, cognitive skills (inhibitory control; working memory), and task types (acceptability judgement; self-paced reading). For this purpose, we employ two word-order patterns (verb-final vs. verb-initial) of two clausal constructions in Korean—suffixal passive and morphological causative—which contrast pertaining to the mapping between thematic roles and case-marking and the interpretive procedures driven by verbal morphology. We find that, while Korean heritage speakers demonstrate the same kind of acceptability-rating behaviour as monolingual Korean speakers do, their reading-time patterns are notably modulated by construction-specific properties, cognitive skills, and proficiency. This suggests a heritage speaker’s ability and willingness to conduct both parsing routes, induced by linguistic cues in a non-dominant language, which are proportional to the computational complexity involving these cues. Implications of this study are expected to advance our understanding of a learner’s mind for underrepresented languages and populations in the field.

## 1 Introduction

The linguistic processor seeks to reduce cognitive burdens of work under the simultaneous activation of (non-)linguistic sources and cognitive–psychological mechanisms (Gibson, 1998; McElree, 2000; Hawkins, 2004; McRae and Matsuki, 2009; Jaeger and Tily, 2011; Traxler, 2014; O’Grady, 2015). Given this nature, the processor’s operation is adjusted by various factors, including language-usage experience (Wells et al., 2009; Jegerski, 2018), grammatical properties of a target item (Dillon et al., 2013; Paolazzi et al., 2019), cue competition (MacWhinney, 1987; Park and Kim, 2022), cognitive skills (Pozzan and Trueswell, 2016; Cunnings, 2017), task types (Tan and Foltz, 2020; Dempsey et al., 2023), and individual differences (Dąbrowska and Street, 2006; Van Dyke et al., 2014).

### 1.1 Good-enough processing in sentence comprehension

The good-enough processing account (GE) reasonably explains how the processor (erroneously) operates during language activities. It assumes two processing routes: an algorithmic stream, which is a structure-based, bottom-up route, and a heuristic stream, which is a usage/experience-based, top-down route (Ferreira, 2003; Christianson, 2016). While these routes apply simultaneously to interpretation, they are distinctive concerning the trade-off between accuracy and efficiency. Algorithms yield precise computations of linguistic representations but require effortful and time-consuming processing. In contrast, heuristics allow rapid and less effortful, yet sometimes underspecified, interpretation. GE maintains that heuristics can generate interpretations earlier than algorithms and that the former sometimes triumphs over the latter. This argument finds support in Ferreira (2003): monolingual English speakers occasionally misinterpret passives (e.g., *The dog was bitten by the man*) by incorrectly mapping thematic roles onto event participants (i.e., an agent role to *the dog* and a theme role to *the man*). This indicates a speaker’s primary commitment to simple, coarse-grained schemata from language-usage experience or world knowledge involving argument realisation in English.

The abovementioned argument is supported by various models/frameworks of sentence processing. Real-time processing places heavy demands on cognitive resources (Gibson, 1998; Lewis et al., 2006; Pozzan and Trueswell, 2015). Therefore, the processor both attempts to immediately finish interpreting input and seeks to avoid repairing misinterpretations unless urgently required (Fodor and Inoue, 1994; Piantadosi et al., 2012). Moreover, because linguistic cues are often noisy (Futrell and Levy, 2017; Gibson et al., 2019) and lossy (Christiansen and Chater, 2016), the processor often settles for options readily accessible from memory (e.g., Noun–Verb–Noun template in English; Townsend and Bever, 2001), provided that these options reasonably preserve communicative intent (Jaeger and Tily, 2011; Kleinschmidt and Jaeger, 2015). This way, the processor achieves and maintains sufficient cognitive equilibrium while minimising burdens on cognitive systems (Kool et al., 2010; Christie and Schrater, 2015; Karimi and Ferreira, 2016).

Much work revolving around GE suggests that the processor prioritises the heuristic route while selectively adopting the algorithmic route when required (Dwivedi, 2013; Kharkwal and Stromswold, 2014; Lim and Christianson, 2015; Qian et al., 2018; Tan and Foltz, 2020), aligning well with the multi-stream models of sentence processing (van Herten et al., 2006; Kuperberg, 2007). However, the literature has predominantly focused on a limited range of languages and speakers of those languages, especially (L2-)English-speaking populations, with few studies exploring beyond this scope (e.g., Russian: Stoops et al., 2014; Mandarin: Zhou et al., 2018). This perpetuates an English-centric perspective (cf. Blasi et al., 2022). Consequently, it is questionable whether previous findings on sentence-processing patterns under GE are generalisable to speakers of lesser-studied languages and usage contexts. Incorporating understudied languages and usage contexts can enrich GE by introducing diverse linguistic structures and language-specific adaptations not commonly found in major languages, thereby challenging and refining our current understanding of this framework.

### 1.2 Working memory and inhibitory control

The relationship between linguistic knowledge and domain-general mechanisms has been actively explored in efforts to address language behaviour (McElree, 2000; Phillips and Ehrenhofer, 2015; Pozzan and Trueswell, 2015; Christiansen and Chater, 2016; Cunnings, 2017). Of various mechanisms, working memory (WM) and inhibitory control (IC) are the major cognitive skills widely discussed in the field. WM refers to a mental workspace that retains information for a short period while concurrently conducting mental operations on this information (Baddeley and Hitch, 1974; Daneman and Merikle, 1996). Although its capacity is limited and varies by individual, it reliably predicts performance in various cognitive tasks, including language comprehension (Tokowicz et al., 2004; Huettig and Janse, 2016; Litcofsky et al., 2016). IC pertains to attentional control for natural, habitual, or dominant responses to a target stimulus that are unnecessary for this stimulus, allowing individuals to become goal-relevant (Miyake and Friedman, 2012; Diamond, 2013). There is growing evidence on the role of IC in language activities (Abutalebi and Green, 2007; Pérez et al., 2020), particularly from bilingualism research: given the parallel activation of both languages, bilinguals utilise IC to attend to one language system while suppressing the other and manage conflicts and interference between the two (Bialystok et al., 2005; Kroll et al., 2008; Wigdorowitz et al., 2023).

WM and IC have routinely been associated with comprehension (Pérez et al., 2014; Tarchi et al., 2021). Most cognitive tasks require both skills (Diamond, 2013), which are intertwined when humans conduct these tasks (Carlson et al., 2002; Roncadin et al., 2007). Each skill, however, seems to differentially affect how language users engage in language activities (Abutalebi and Green, 2007; Linck and Weiss, 2015). This stems from their representation of varying aspects of cognitive skills (Astle et al., 2013)—even though they outwardly share some fundamental architectures (Miyake et al., 2000)—and their asymmetric changes across a lifespan (Williams et al., 1999; Robert et al., 2009).

### 1.3 Target population: Korean heritage speakers

Heritage speakers are defined as child and adult members of a linguistic minority whose home language involves limited usage experience and formal literacy education in a community and the majority language in that community is dominantly used (Rothman, 2009; Montrul, 2010). They manifest asymmetric linguistic representations influenced by various factors such as reduced home-language input, pressure on usage from the majority language, grammatical properties of a target item, and cognitive resources (Kondo–Brown, 2005; O’Grady et al., 2011; Jia and Paradis, 2015; Mikhaylova, 2018; Felser and Arslan, 2019; Karaca et al., 2024; for an in-depth overview, see Polinsky and Scontras, 2020). Previous studies have delineated distinctive attributes of heritage speakers’ morphosyntactic knowledge in comparison to monolingual or L2 speakers (Kim et al., 2009; Laleko and Polinsky, 2016; Montrul et al., 2019; Fuchs, 2022). Furthermore, research has elucidated the role of individual differences (represented as WM, IC, or overall proficiency in the heritage language) in modulating heritage speakers’ task performance (Chondrogianni and Schwartz, 2020; Bice and Kroll, 2021; Torres, 2023). Exploring the language-processing mechanisms of heritage speakers is crucial not only for unveiling the unique challenges stemming from their language-use backgrounds but also for enlightening researchers and practitioners to better understand and empower this population in preserving their heritage language.

Our study specifically focuses on Korean heritage speakers (KHSs) residing in the United States. With over 1.9 million individuals speaking Korean as a heritage or community language in the country, this demographic constitutes the fifth-largest Asian-American subgroup (U.S. Census Bureau, 2021). Despite the increasing global interest in Korean culture and language, research within the US contexts has predominantly centred on dominant heritage speaker groups such as Hispanics or Chinese (Jegerski et al., 2016; Scontras et al., 2017; Hur et al., 2020; Bice and Kroll, 2021; Torres, 2023; López Otero et al., 2024), underscoring the urgent need for scholarly attention towards KHSs. Korean, an understudied language for GE, is a Subject–Object–Verb language that maintains verb-finality, but its case-marking system allows for relatively free word order by scrambling sentential components (Sohn, 1999). We concentrate on two clausal constructions—suffixal passive and morphological causative—which contrast with respect to alignments between thematic roles and case markers as well as interpretive procedures involving verbal morphology.

In this study, we investigate how KHSs engage in sentence comprehension under GE, serving as the basic sentence-processing architecture, with a focus on the two parsing streams situated at the intersection of proficiency (as an indicator of home language usage experience), cognitive skills, and task types. Regarding task modalities, we examine two language tasks: acceptability judgement and self-paced reading. In an acceptability judgement task (AJT), a comprehender partially and holistically evaluates a sentence under few time constraints before arriving at a complete interpretation of the sentence and decides on its acceptability. A self-paced reading task (SPRT) requires a comprehender to conduct moment-by-moment, sequential, and cumulative interpretations of incoming items in real time, subject to stricter time constraints compared to AJT. Given the commonly observed disparities between non-dominant-language learners’ explicit and implicit/automatised knowledge (Jiang, 2007), some studies have explored how varying task demands affect the GE processing of L2-English sentences (translation vs. eye-tracking: Lim and Christianson, 2015; comprehension question types during SPRT: Tan and Foltz, 2020). However, research on this topic for underrepresented languages and populations remains less active.

## 2 Study 1: suffixal passive construction

### 2.1 Linguistic descriptions of the target construction

The passive voice is marked across languages (Haspelmath, 1990; Siewierska, 2013), and its usage frequency in Korean is notably lower than that of the active voice (Woo, 1997; Park, 2021). The suffixal passive consists of two arguments, a nominative-marked theme subject and a dative-marked agent oblique, followed by a passivised verb. Passive morphology, signaled by one of the four allomorphic variants of verbal suffixes (*-i/hi/li/ki-*), serves as a key disambiguation point for identifying a sentence’s structural properties, also decreasing the verb’s valency slots (from two to one for a transitive verb). The canonical pattern (1a) follows the theme–agent–verb sequence, but the verb can be fronted via scrambling, yielding a verb-initial pattern (1b) found in colloquial speech for afterthought clarification, information amplification, or emphasis (Sohn, 1999).

**Table:** 

(1)	Korean suffixal passive: “The thief was caught by the police.”		
a.	Verb-final		
	Totwuk-i kyengchal-hanthey		cap-hi-ess-ta.
	thief-NOM police-DAT		catch-PSV-PST-SE
b.	Verb-initial		
	Cap-hi-ess-ta totwuk-i		kyengchal-hanthey.
	catch-PSV-PST-SE thief-NOM		police-DAT

The two word-order patterns exhibit contrastive characteristics regarding the timing of disambiguation. In the verb-final pattern (1a), passive morphology constitutes a late-arriving cue, compelling a comprehender to revise an initial analysis conducted before encountering this morphology. In Korean, a nominative-marked [+human] argument is likely to be interpreted as an agent, and a dative-marked [+human] argument tends to be interpreted as a recipient. These interpretations are supported by strong associations between thematic roles and case markers attested in language use (Sohn, 1999; Kim and Choi, 2004; Shin and Mun, 2023). Therefore, a plausible way of analysing (1a) prior to the verb is that the thief executes an action affecting the police. However, this analysis is incongruent with the passive-voice information conveyed by verbal morphology. Upon encountering the sentence-final verb, a comprehender must revise their initial interpretation, recalibrating the arguments’ thematic roles by mapping a theme role onto the nominative-marked entity and an agent role onto the dative-marked entity. Revision in this manner is linguistically and cognitively demanding (Rapp and Kendeou, 2007; Kendeou et al., 2013), thereby posing challenges to language activities (Kim et al., 2017; Shin, 2022). The situation differs for the verb-initial pattern (1b). The fronted verb and its morphology constitute an early-arriving cue, guiding the succeeding interpretation of the arguments and forestalling possible misinterpretations of these arguments’ thematic roles (cf. Pozzan and Trueswell, 2015). Therefore, the sentence-initial verb in (1b) is expected to inform a comprehender that the nominative-marked entity *thief* is not the agent but the theme and that the dative-marked entity *police* is not the recipient but the agent, also suppressing the typical associations between thematic roles and case markers.

### 2.2 Methods: study 1^1^

#### 2.2.1 Participants

We recruited 40 KHSs (*M*_*age*_ = 24.0, SD = 5.2) who were born in the USA, were raised by Korean-speaking parents, and had resided in the USA for most of their lives (length of stay in the USA: M = 21.9, SD = 6.2). They used English more frequently than Korean in daily life (English: M = 92.5, SD = 9.5; Korean: M = 37.1, SD = 27.3; score out of 100) and adopted Korean more often with family than colleagues (family: M = 4.98, SD = 1.25; friends: M = 3.45, SD = 1.38; colleagues: M = 3.25, SD = 1.63; score out of 6 [1 = English only; 6 = Korean only]). They expressed greater confidence in their proficiency in listening to and speaking Korean (M = 4.03, SD = 0.92 [0 = not good; 5 = very good]) compared to their skills in reading and writing Korean (M = 3.05, SD = 1.20 [0 = not good; 5 = very good]), also confirmed by a one-sample *t*-test: *t*(78) = 4.085, *p* < 0.001. Nevertheless, they expressed dissatisfaction with their ability to speak Korean (M = 2.83, SD = 1.39 [0 = not satisfied; 5 = very satisfied]) and perceived their command of Korean as falling short of target-like use (M = 2.05, SD = 1.66 [0 = fully disagree; 5 = fully agree]). All the KHSs in this study learnt Korean primarily from their parents, supplemented by additional exposure through three major channels: educational institutions such as language schools, universities, and academies (80%), online resources (70%), and social interactions with friends and peers (70%). We also recruited 32 monolingual speakers of Korean (MSK; *M*_*age*_ = 25.7, SD = 4.3) as a control group.

#### 2.2.2 Materials, procedures, and analysis

Participants joined a *Zoom* meeting and completed the tasks individually on web-based platforms: proficiency (JavaScript-based), cognitive task (*PsyToolkit*; Stoet, 2010, 2017), SPRT (*PCIbex*^2^; Zehr and Schwarz, 2018), AJT (*Qualtrics*), and background survey (*Google Forms*). For the stability of testing environments, mobile devices were prohibited. Participants completed the tasks at their convenience provided that they could maintain a good internet connection and focus on the activities. We asked them to (i) check their internet connection and clear their surroundings before starting the tasks and (ii) stay in the meeting room until all tasks have been completed for us to observe their participation. Test sentences are illustrated in Supplementary Appendix A.

##### 2.2.2.1 Proficiency

Proficiency in Korean was measured through the Korean C-test (Lee-Ellis, 2009), which involves the comprehension of Korean sentences of varying lengths and complexities. It consists of five passages containing blanks at the syllable level. Each blank represents a syllable from either a content or function word and may appear in various positions within an eojeol (a white-space-based segment serving as a minimal language unit in Korean). We chose the first four passages for testing efficiency, as suggested by the original study. Although construction types were not the primary focus during the development of this test in Lee-Ellis (2009), a manual examination of the four passages confirmed that none of the targeted constructions in the current study appeared. The representative sentence structures used in the test included (in)transitive constructions, locative constructions, double-nominative constructions, coordinate/subordinate clauses, and relative clauses, with scrambling and omission of sentential components involved. Each blank corresponded to one point, and the maximum score possible was 188. The proficiency scores of participants (M = 127.3, SD = 25.8) exceeded those of L2 learners in Lee et al. (2023), indicating that KHS possessed commendable literacy and reading skills in their home language.

##### 2.2.2.2 Cognitive task

We measured participants’ WM via a digit-span task (Miller, 1956) considering its popularity in the field, simplicity of implementation and interpretation, and superiority to other measurement types (Schofield and Ashman, 1986; Baddeley et al., 1998; Wechsler, 2009; Jones and Macken, 2015). Participants were exposed to a sequence of two digits. A longer sequence was presented if they succeeded in repeating the sequence and until they failed to repeat it correctly. The longest sequence that they retrieved correctly was considered their digit span. To ensure this measure’s reliability, sequence length was increased after recall of the same length twice.

We also measured participants’ IC by employing an open-source version (provided by *PsyToolkit*) that slightly adapted the original Flanker task developed by Eriksen and Eriksen (1974). Participants were presented with five letters and instructed to respond to the one in the middle by pressing “A” on the keyboard when they saw “X” or “C” and pressing “L” upon perceiving “V” or “B.” We counted the total number of correct responses out of 50 trials, whether under congruent (i.e., target = flank [letters surrounding the target]) or incongruent (i.e., target ≠ flank) conditions, and excluded excessively slow responses (whose reaction time was above 3,000 ms). Each task lasted for around five minutes.

##### 2.2.2.3 Self-paced reading task

We created 16 Korean suffixal passive sentences (verb-final: eight; verb-initial: eight),^3^ each comprising a carrier phrase (e.g., *Nay-ka tul-ess-nuntey*, “I heard that”), followed by the critical passive structure (verb-final: theme–agent–verb; verb-initial: verb–theme–agent) and a temporal adverbial phrase consisting of two words (e.g., *ecey pam-ey*, “last night”), as in (2a–b). For agent/theme nominals, we used human names often attested in daily life. All the verbs (with sufficient usage frequency) were expressed in the past tense, and no overlap occurred in verb use across the sentences in each condition. The sentences were counterbalanced for the two conditions across two lists, and each participant encountered only one condition of a single item. During the task, each item was presented in six regions (Rs), with R2, R3, and R4 as the main regions of interest and R5 as an additional region for accommodating the spill-over effects induced by a task-specific button-press strategy (Koornneef and Van Berkum, 2006). The test sentences were interspersed with 48 fillers of various structures and complexities.

**Table:** 

(2)	Example of stimuli: “I heard that Mia was hugged by Pola last night.”	
a.	Verb-final	
	[Nay-ka tul-ess-nuntey]_*R1*_ [Mia-ka]_*R2*_ [Pola-hanthey]_*R3*_
	I-NOM hear-PST-COMP Mia-NOM Pola-DAT
	[an-ki-ess-tay]_*R4*_ [ecey]_*R5*_ [pam-ey.]_*R6*_
	hug-PSV-PST-SE yesterday night-TIME
b.	Verb-initial		
	[Nay-ka tul-ess-nuntey]_*R1*_ [an-ki-ess-tay]_*R2*_ [Mia-ka]_*R3*_
	I-NOM hear-PST-COMP hug-PSV-PST-SE Mia-NOM
	[Pola-hanthey]_*R4*_ [ecey]_*R5*_ [pam-ey.]_*R6*_
	Pola-DAT yesterday night-TIME

Prior to the experiment, we conducted a norming task to assess Korean speakers’ general acceptance of the test sentences. Because we presented the verb-initial sentences without any context promoting scrambling, we ensured these sentences to be accepted as grammatical, albeit to a lesser degree than the verb-final counterparts. Ten monolingual Korean speakers who did not join the experiment evaluated the sentences’ grammaticality using a binary scale (grammatical [1], ungrammatical [0]). The mean acceptability rates were 100% for the verb-final sentences and 93.8% for the verb-initial sentences, indicating that the speakers regarded the sentences in both conditions as grammatical. These inspectors reported that they sometimes rejected the verb-initial sentences because they had a lower preference for scrambled sentences out of context and not because of the difference in grammaticality between the two conditions.

SPRT was run under a non-cumulative moving-window paradigm (Just et al., 1982), with each target sentence appearing at the centre of the screen on a region-by-region basis. In the beginning of each trial, participants saw a series of dashes on-screen, and each press of a spacebar revealed words in each region while concealing preceding words. Following each sentence, a simple comprehension question appeared to direct participants’ attention to the task. Participants responded by clicking on one of two choices, and upon the choice of an erroneous answer, a “wrong choice” feedback appeared on-screen. Each question involved simple facts regarding the sentence being read (e.g., what the sentence was about, what action was done), in contrast to previous studies wherein questions asked about an agent or a theme and answers served as reflections of comprehenders’ misunderstanding (e.g., Ferreira, 2003). We used participants’ responses only as an attention check (cf. Dwivedi, 2013). Prior to the experiment, they received written instructions and worked through three practice items for familiarisation with the procedures. The task took approximately 20 minutes.

Data from SPRT were first trimmed by excluding the reading time (RT) datapoints of all the regions in a sentence upon failure in the comprehension check for that sentence (data loss: 1.06%) and by excluding outliers per region through a three-standard-deviation cut-off point (collapsing over item and participant; data loss: 2.94%). We then log-transformed the pruned data for normalisation and residualised them to adjust for variability in word length and individual reading speed (Baayen and Milin, 2010). For the residualisation (following Trueswell et al., 1994), we first obtained predicted RT estimates for each participant (including both MSK and KHS groups) across all experimental trials computed based on a linear mixed-effects model with word length (i.e., number of syllables) in a region as a fixed effect and Participant as a random effect. We then calculated residual RTs by subtracting the predicted RTs from the log-transformed RTs for each participant. The pre-processed data were fitted to linear mixed-effect modeling for each critical and spill-over region, with *Group* and *Condition* as fixed effects (centred around the mean and deviation-coded) and with *Participant* and *Word* as random effects using *lme4* (Bates et al., 2015) in R (R Core Team, 2023). The models included the maximal random-effects structure allowed by modeling with random intercepts and slopes for all effects (Barr et al., 2013). For KHS-internal models, the three factors (*Digit*, *Flanker*, *Proficiency*) were treated as continuous variables and employed as fixed effects; each model consisted of only two fixed effects (*Condition* and one of these factors), resulting in three sub-models per region. The other specifications were the same as those in the global model.

##### 2.2.2.4 Acceptability judgement task

The sentences for AJT were created by clipping the main regions of test sentences used in SPRT. Only one sentence appeared on the screen per trial. Participants were instructed to rate the acceptability of each sentence with a 6-point Likert scale (unacceptable: 0; acceptable: 5), responding immediately upon encountering the sentence but without sacrificing the accuracy and faithfulness of/confidence in their response. Once participants clicked on the scale and moved on to the next sentence, they were prohibited from revising their previous evaluation. This task was untimed and took approximately 15 minutes.

Data from AJT were trimmed by excluding the individual values with response times below 1,000 ms or above 10,000 ms (data loss: 5.55%). We then *Z*-transformed the pruned data for normalisation and proceeded to the same kind of linear mixed-effect modeling, with *Group* and *Condition* as fixed effects (centred around the mean and deviation-coded) and with *Participant* and *Sentence* as random effects (Bates et al., 2015) in R (R Core Team, 2023), including the same maximal random-effects structure as that in the SPRT data analysis (Barr et al., 2013). For KHS-internal models, the three factors (*Digit*, *Flanker*, *Proficiency*) were treated as continuous variables and employed as fixed effects; each model consisted of only two fixed effects (*Condition* and one of these factors), resulting in three sub-models per region. The other specifications were the same as those in the global model.

### 2.3 Predictions: study 1

#### 2.3.1 Monolingual speakers of Korean

In SPRT, we anticipate no substantial RT difference between the two conditions. This is attributed to the primary adoption of the heuristic-before-algorithm strategy by monolingual speakers (Ferreira, 2003; Dwivedi, 2013; Kharkwal and Stromswold, 2014; Christianson, 2016; Lee et al., 2023) favouring the canonicity/typicality involving word order (heuristic parsing) over interpretive procedures involving passive morphology (algorithmic parsing), coupled with their adept utilisation of both parsing routes. Similarly, in AJT, we anticipate that MSK will rate the verb-final condition as more acceptable than its verb-initial counterpart. This arises from the infrequent and less plausible/felicitous nature of scrambling in the absence of contextual cues that typically license such syntactic rearrangements.

#### 2.3.2 Korean heritage speakers

In SPRT, we predict three general outcomes of KHS’s processing of the two patterns. First, more time would be spent reading sentences underlain by both patterns than that spent by MSK given the global difficulty in the real-time processing of non-dominant languages (McDonald, 2006; Hopp, 2014; Grüter and Rohde, 2021) and the reduced degree/richness of home-language exposure (Unsworth, 2013; Jia and Paradis, 2015). Second, RT would increase at/after the verb in both patterns due to the interpretive procedures involving passive morphology. Third, the RTs between the verb regions of the two conditions would be comparable, owing to the competing dynamics of heightened surprisal/disequilibrium associated with verb-initiality at R2 in the verb-initial condition versus the interpretive support by verb-finality (i.e., canonicity of word order) in handling the necessary revision process at R4 in the verb-final condition. Pertaining to the three factors (WM, IC, proficiency), we expect the degree to which they influence KHSs’ RT patterns to differ. The sentence-initial verb is atypical and less expected than a nominative-marked noun in sentence composition, potentially generating more surprisal/disequilibrium in the verb-initial pattern than its verb-final equivalent. This situation can be controlled via IC, efficiently suppressing interference from the competitor of the verb-initial pattern (i.e., its verb-final counterpart). Therefore, we would see decreased RTs when handling the fronted verb proportionate to IC capacities. The sentence-final verb is typical in sentence composition, but passive morphology necessitates the revision of the initial mapping between thematic roles and case markers. WM would help retrieve a previous interpretation and conduct the required procedure efficiently, thus reducing RTs when handling the sentence-final verb proportionate to large WM capacities. Proficiency would then ensure the overall efficiency with which a non-dominant language is processed, resulting in KHSs’ decreased RTs over the entire structure for both patterns.

In AJT, KHS would accept the verb-final pattern more than its verb-initial counterpart, similar to MSK and consistent with prior studies showing heritage speakers’ better performance on the canonical word order in comparison to the non-canonical word order (Kim et al., 2018; Chondrogianni and Schwartz, 2020). This stems from two promising forces. One is the typicality of sentence composition in Korean—subject-first and predicate-final, which is frequent and readily available/accessible from memory. The other is the task’s introduction of scrambled sentences, often accompanying contextual/discoursal effects without contextual lead-ins. These forces would increase the acceptability of the verb-final pattern over the verb-initial pattern. We also anticipate the rating gap to expand (i.e., evaluating the verb-initial pattern to be lower) as proficiency or cognitive skills increase because these factors would strengthen KHSs’ recognition of the unnaturalness of scrambled sentences in the experiment.

### 2.4 Results: study 1

#### 2.4.1 Cognitive skills

For the digit-span task, the mean score of KHS was 6.6 (SD = 1.3). When compared to that of MSK (M = 7.8, SD = 1.1), the two groups differed (independent-sample *t*-test: *t*(70) = –4.062, *p* < 0.001). For the Flanker task, the mean score of KHS was 42.3 (SD = 6.3). When compared to that of MSK (M = 38.9, SD = 11.8), the two groups did not differ (independent-sample *t*-test: *t*(70) = 1.552, *p* = 0.125).

#### 2.4.2 Self-paced reading

**FIGURE 1:**
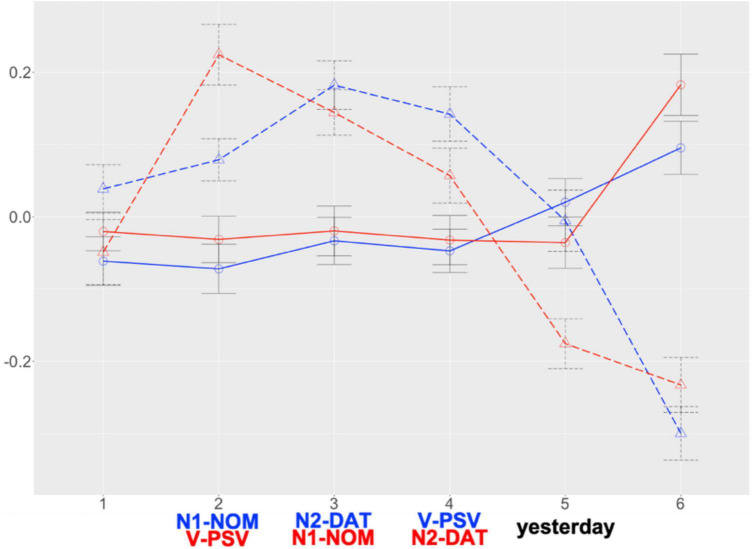
Results: Suffixal passive (SPRT). *X*-axis: region; *Y*-axis: residual RT. Blue: verb-final; Red: verb-initial; solid line: MSK; dashed line: KHS. Error bars: 95% CI.

**FIGURE 2:**
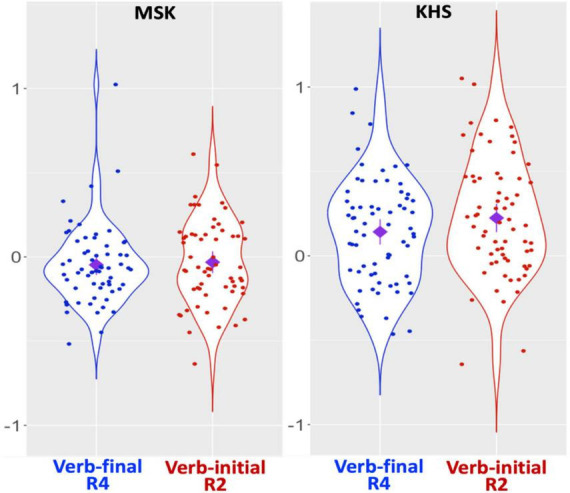
Results: Suffixal passive (SPRT), verb regions only. *X*-axis: condition; *Y*-axis: residual RT. Diamond: mean; Error bars: 95% CI.

Figure 1 presents the two groups’ RT patterns (see Supplementary Appendix B, Supplementary Table A for raw RTs, log-transformed RTs, and residualised RTs per region) and Figure 2 illustrates their RT patterns focusing on verb regions. The global model (Supplementary Appendix B, Supplementary Table B) revealed main effects of *Group* at all the regions of interest and *Condition* at R2 and R5. Additional analyses (α = 0.025) showed no difference at each region for MSK but significant differences at R2 (β = 0.146, SE = 0.047, *t* = 3.098, *p* = 0.002) and R5 (β = –0.170, SE = 0.054, *t* = –3.157, *p* = 0.002) for KHS. These indicate that, given the overall by-group difference (R2 to R4: KHS > MSK; R5: KHS < MSK), KHS demonstrated notable by-condition variances (R2: verb-final < verb-initial; R5: verb-final > verb-initial). A verb-region model (fixed effects: *Group*, *Condition*; random effect: *Participant* only due to model convergence issues; α = 0.025; *R*^2^ = 0.206) further revealed only a main effect of *Group* (β = 0.225, SE = 0.040, *t* = 5.652, *p* < 0.0005), indicating that each group spent comparable RTs across the two verb regions.

**FIGURE 3:**
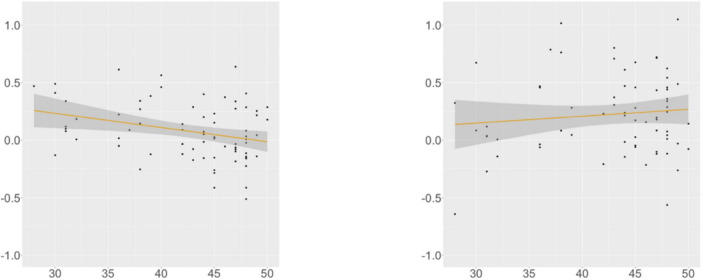
Suffixal passive, KHS, R2, Flanker (*x*-axis) ∼ residual RT (*y*-axis). Gray areas: 95% CIs. Left: verb-final (*r* = −0.301, *p* = 0.009); Right: verb-initial (*r* = 0.109, *p* = 0.377).

KHS-internal models (Supplementary Appendix B, Supplementary Tables C–E) revealed that each factor (*Digit*, *Flanker*, *Proficiency*) differentially contributed to the models. At R2, we found an interaction effect between *Condition* and *Flanker*, and post-hoc analyses (α = 0.0125) uncovered marginal significance in the verb-final condition (β = –0.012, SE = 0.005, *t* = –2.455, *p* = 0.018) and insignificance in the verb-initial condition. This trend was supported by the correlation analysis (Figure 3), in which the association between the Flanker scores and the RTs was meaningful only in the verb-final condition. These indicate that KHS spent less time reading R2 in the verb-final condition as their IC capacities expanded.

**FIGURE 4:**
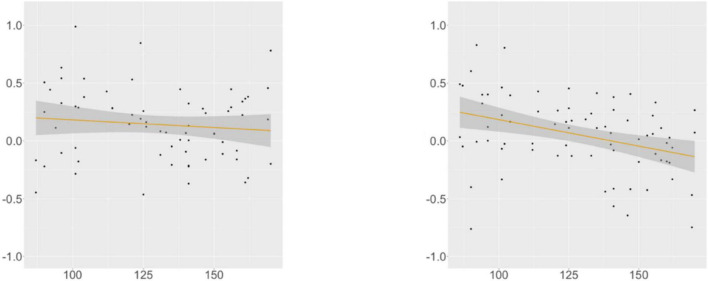
Suffixal passive, KHS, R4, proficiency (*x*-axis) ∼ residual RT (*y*-axis). Gray areas: 95% CIs. Left: verb-final (*r* = −0.107, *p* = 0.384); Right: verb-initial (*r* = −0.361, *p* = 0.001).

At R4, we found a main effect of *Proficiency*; additional analyses (α = 0.0125) yielded insignificance in the verb-final condition and significance in the verb-initial condition (β = –0.004, SE = 0.001, *t* = –3.354, *p* = 0.001). This trend was supported by the correlation analysis (Figure 4), with the association between the proficiency scores and the RTs being meaningful only in the verb-initial condition. These indicate that, given the broad impact of proficiency on the RTs at this region, KHS spent less time particularly in the verb-initial condition with increasing proficiency.

**FIGURE 5:**
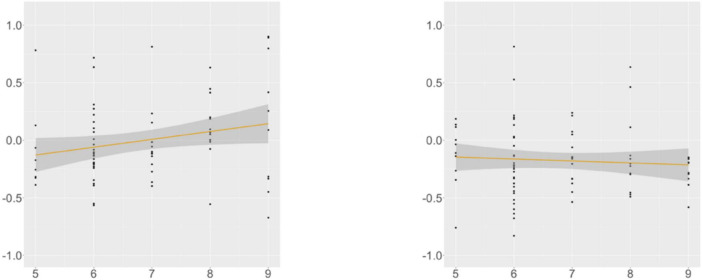
Suffixal passive, KHS, R5, digit (*x*-axis) ∼ residual RT (*y*-axis). Gray areas: 95% CIs. Left: verb-final (*r* = 0.231, *p* = 0.047); Right: verb-initial (*r* = −0.070, *p* = 0.543).

At R5, we found a marginal interaction effect between *Condition* and *Digit*, and additional analyses (α = 0.0125) yielded insignificance in both conditions. However, a meaningful relationship existed between the digit-span scores and the RTs in the verb-final condition, as shown by the correlation analysis (Figure 5). These indicate that KHS spent more time reading R5 in the verb-final condition with larger (albeit weak) WM capacities.

Neither of verb-region models (fixed effects: *Condition* and one of the following factors [*Digit*, *Flanker*, *Proficiency*]; random effect: *Participant* only due to model convergence issues; α = 0.025) revealed significant main or interaction effects (all *p*s > 0.1).

#### 2.4.3 Acceptability judgement

**FIGURE 6:**
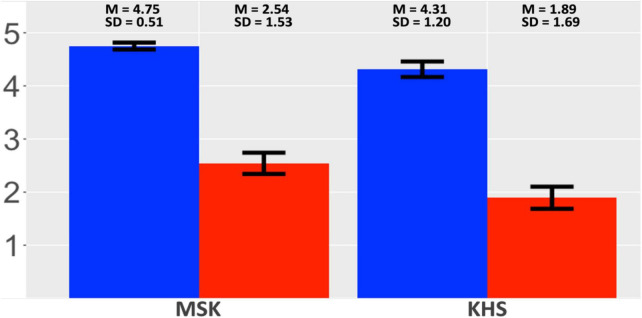
Results: Suffixal passive (AJT). *X*-axis: Group; *Y*-axis: acceptability. Blue: verb-final; red: verb-initial. Error bars: 95% CI.

Figure 6 presents the two groups’ acceptability-rating outcomes. Both groups rated the verb-final condition to be more acceptable than the verb-initial condition, but the by-condition gap was larger for KHS than MSK. The global model (Supplementary Appendix B, Supplementary Table F) revealed a main effect of *Condition* and an interaction effect between *Condition* and *Group*. Post-hoc analysis (α = 0.025) yielded insignificance for all the by-group comparisons, indicating uniformity in the two groups’ preference for the verb-final condition.

KHS-internal models (Supplementary Appendix B, Supplementary Tables G–I) revealed interaction effects between *Condition* and each factor (*Digit*, *Flanker*, *Proficiency*), indicating that KHS evaluated the verb-initial condition as less acceptable with increasing WM capacities, IC capacities, or proficiency.

### 2.5 Discussion: study 1

The results on the two tasks performed by MSK are consistent in light of how the two parsing streams operate. Together with the higher acceptability ratings for the verb-final condition than the verb-initial condition in AJT, no processing benefit was derived from the fronted passive morphology in SPRT, as predicted. These suggest a larger role of heuristics (word-order typicality) than algorithms (interpretive procedures driven by verbal morphology) in processing the suffixal passive, aligning with previous studies (Ferreira, 2003; Dwivedi, 2013; Kharkwal and Stromswold, 2014).

The performance of KHS in the two tasks elucidates how GE operates in conjunction with various factors during comprehension. While exhibiting similar acceptability-rating behaviour to MSK, KHS demonstrated prolonged RTs at the critical regions (R2–R4) in both conditions, as predicted. This aligns with prior research that highlights a general challenge in the real-time processing of non-dominant languages (Pozzan and Trueswell, 2016; Tachihara and Goldberg, 2020; Grüter and Rohde, 2021). The fronted verb in the verb-initial condition incurred greater processing cost compared to the nominative-marked subject in the verb-final condition, whereas the post-verbal region in the verb-initial condition incurred reduced processing cost compared to that region in the verb-final condition. Importantly, KHS exhibited similar RTs when reading the verb region in both conditions, consistent with our prediction. These findings indicate a lack of evidence for the active role of early-appearing verbal morphology cues in processing verb-initial passive sentences. That is, the presumed advantage of fronted verbal morphology may not have completely surpassed the processing benefit of the canonical word order which is readily accessible from memory (and may potentially alleviate the interpretive complexity posed by passive morphology occurring at the end of a sentence). This is ascribable to multiple possibilities: heritage speakers’ susceptibility to home-language morphosyntax (Laleko and Polinsky, 2016; Kim et al., 2018; Chondrogianni and Schwartz, 2020), their reduced flexibility in dealing with scrambling due to dominant language—English in this study (Namboodiripad et al., 2018), and limited usage experience of home language (Chondrogianni and Schwartz, 2020; Hur et al., 2020; López Otero et al., 2024).

Notably, KHS’s performance was modulated by cognitive skills and proficiency. This interplay was more complex in SPRT than AJT: the KHS’s acceptability ratings were proportionate to their scores on the three measures (digit span, Flanker, proficiency), but the contributions of these measures to their RT patterns varied at different regions and conditions, which deviated from our predictions. KHS spent less time reading R2 in the verb-final condition with expanding IC capacities, but this trend was missing from the verb-initial condition. This difference is ascribed to an increased degree of interpretive challenge involving verb-initiality. That is, the fronted verb—manifesting atypical word order and inviting (re)calibrations of the mapping between thematic roles and case markers early on—may have substantially canceled out processing support from IC when KHS handled the early-appearing verb/morphology cue.

KHS spent less time reading R4 in the verb-initial condition with increasing proficiency. This can be interpreted in two ways. First, it suggests KHS’s larger space for, and more engagement in, handling passive morphology (and algorithmic parsing tied to that morphology) in the verb-final condition as proficiency increased. Second, it implies KHS’s enhanced efficiency in processing a dative-marked agent in the verb-initial condition as proficiency increased. Meanwhile, the expected role of proficiency over the entire structure did not emerge. This finding contradicts previous research demonstrating the facilitative role of general language proficiency in a non-dominant language for achieving target-like processing of clausal constructions (Jackson, 2008; Rah and Adone, 2010; Kaan, 2014; Hopp, 2017). Rather, this finding lends support to the idea that proficiency in the target language (as one factor of individual variability) selectively influences sentence processing within that language contingent upon task types (Roberts, 2012).

KHS spent more time reading R5 (spill-over involving clausal integration for complete interpretation) in the verb-final condition as their WM capacities expanded although the effect was weak. This finding is the reverse of our prediction, implying KHS’s increased capacity for, and commitment to, the integration procedures involving the canonical word-order condition proportionate to their WM skills. Such capacity and commitment, in turn, enable a comprehender to reserve more space for coping with previous and current inputs at this region.

Nevertheless, it is premature to draw firm conclusions about the operational characteristics of the two parsing routes solely from Study 1. The fact that each group spent comparable RTs across the two verb regions (as shown in Figure 2) might also imply the possibility that heuristic parsing was counterbalanced by algorithmic parsing. Hence, additional evidence is required to convincingly elucidate the interplay between KHS’s sentence-processing behaviour and various factors within GE, particularly concerning the heuristic-before-algorithm strategy. We thus conducted another experiment, focusing on the morphological causative manifesting interpretive procedures involving verbal morphology distinctive from those of the suffixal passive.

## 3 Study 2: morphological causative construction

### 3.1 Linguistic descriptions of the target construction

The morphological causative consists of three arguments: a subject (causer), an indirect object (causee), and a direct object (theme), as in (3a). The verb carries one of the seven allomorphic variants of verbal suffixes (*-i/hi/li/ki/wu/kwu/chwu-*), thereby increasing the verb’s valency slots. The verb can move to the sentence-initial position as in (3b).

**Table:** 

(3)	Korean morphological causative: “Mia made Pola eat food.”
a.	Verb-final
	Mia-ka Pola-eykey umsik-ul mek-i-ess-ta.
	Mia-NOM Pola-DAT food-ACC eat-CST-PST-SE
b.	Verb-initial
	Mek-i-ess-ta Mia-ka Pola-eykey umsik-ul.
	eat-CST-PST-SE Mia-NOM Pola-DAT food-ACC

The interpretation of the arguments’ thematic roles hinges upon causative morphology, but this process does not invoke substantial challenges to the extent that passive morphology does. To illustrate, in (3a), the nominative-marked [+human] argument *Mia-ka* is understood as a causer (as an extension of an agent, sharing the concept of a volitional actor). The dative-marked [+human] argument *Pola-eykey* is understood as a causee (as an extension of a recipient); the dative marker ensures these extensions by sharing the same semantic component—GOAL (Sohn, 1999). Causative morphology does not invite the same kind of recalibration of the mapping between thematic roles and case markers as that needed in passive morphology. Therefore, the degree of cognitive burdens that verbal morphology poses to processing the morphological causative is not enormous.

### 3.2 Methods: study 2

#### 3.2.1 Participants

The same participants in Study 1 joined this experiment a week after their initial participation.

#### 3.2.2 Materials, procedures, and analysis

Participants joined only SPRT and AJT at this time. For SPRT, we created 16 test sentences (verb-final: eight; verb-initial: eight). To make critical and spill-over regions as comparable as possible across Studies 1 and 2, we structured the target sentences analogously to those in Study 1. Specifically, we omitted an accusative case marker of the direct object and topicalised it by moving it to the sentence-initial position as in (4); the target frame (R2–R4) contained a nominative-marked NP, a dative-marked NP, and a verb—a structure that closely paralleled the material in Study 1.^4^

**Table:** 

(4)	Example of stimuli: “That shoe, Mia made Pola wear last night.”
a.	Verb-final
	[Ce sinpal]_*R1*_, [Mia-ka]_*R2*_ [Pola-hanthey]_*R3*_ [sin-ki- ess-tay]_*R4*_
	that shoe Mia-NOM Pola-DAT wear-CST-PST-SE
	[ecey]_*R5*_ [pam-ey.]_*R6*_
	yesterday night-TIME
b.	Verb-initial
	[Ce sinpal]_*R1*_, [sin-ki-ess-tay]_*R2*_ [Mia-ka]_*R3*_ [Pola- hanthey]_*R4*_
	that shoe wear-CST-PST-SE Mia-NOM Pola- DAT
	[ecey]_*R5*_ [pam-ey.]_*R6*_
	yesterday night-TIME

Participants were randomly assigned to one of two lists counterbalanced for the word-order condition. Ten monolingual Korean speakers who did not participate in either Study 1 or 2 evaluated the grammaticality of the test sentences using a binary scale (grammatical; ungrammatical). The mean acceptability ratings were 100% and 93% for sentences in the verb-final and verb-initial conditions, respectively, indicating that the sentences were grammatical. The lower score for the verb-initial than the verb-final condition was due to scrambling without context. The test sentences were intermixed with 48 fillers of various structures and complexities.

For AJT, we crafted sentences using the critical structure portion (verb-final: R2 + R3 + N-ACC + R4; verb-initial: R2 + R3 + R4 + N-ACC) from the test stimuli, together with the fillers, used in SPRT. The clausal composition of the test sentences across the two tasks differed because of the topicalised theme object for SPRT. We acknowledge that it could have been a confound in precisely revealing task effects through this construction.

Data from SPRT were trimmed by excluding incorrect responses to the comprehension check-up questions (data loss: 1.07%) and RTs beyond 3SD from the mean (data loss: 3.13%). Data from AJT were trimmed by excluding individual values whose response times were less than 1,000 ms or more than 10,000 ms (data loss: 6.25%). The trimmed data from each task were analysed in the same manner as in Study 1.

### 3.3 Predictions

#### 3.3.1 Monolingual speakers of Korean

In SPRT, given the primary processing strategy (i.e., heuristic-before-algorithm), if the comparatively less demanding nature of causative morphology (in contrast to passive morphology) influences the monolinguals’ real-time processing behaviours, MSK may exploit the fronted verb (and causative morphology) upon encountering the causative frame. This would lead to reduced RTs in the verb-initial condition compared to the verb-final condition. In AJT, MSK will exhibit higher acceptability ratings in the verb-final condition compared to the verb-initial condition, mirroring the findings of Study 1.

#### 3.3.2 Korean heritage speakers

Causative morphology does not necessitate drastic revisions of the initial interpretation, as is the case with passive morphology. We thus expect that, although KHS would demonstrate the same kind of acceptability-rating trends for the word-order canonicity in AJT and the overall difficulty in the real-time processing of both patterns in SPRT as in Study 1, they would be able to take advantage of the less complex nature of interpretive procedures involving causative morphology to some degree. This would be indicated through KHS’s shorter RTs of verb-related regions in one condition relative to the same regions in the other condition in SPRT. Meanwhile, the comparatively simpler interpretive nature of causative morphology (in contrast to passive morphology) may lead heritage speakers to exhibit a more pronounced reaction to verb-initiality than verb-finality. This could result in heightened surprisal/disequilibrium in the verb-initial condition. If such a scenario occurs, longer RTs would likely be observed at R2 in the verb-initial condition compared to R4 the verb-final condition. Pertaining to the three factors (WM, IC, proficiency), we generally anticipate that the extent to which these factors influence KHS’s RT patterns would differ, as found in Study 1. Specifically, given the less radical (re)alignment between thematic roles and case markers driven by causative morphology than that driven by passive morphology, IC would better advance the management of the surprisal/disequilibrium generated in the verb-initial pattern, efficiently suppressing interference from its competitor. This would be indicated through decreased RTs when coping with the fronted verb proportionate to IC capacities.

### 3.4 Results: study 2

#### 3.4.1 Self-paced reading

**FIGURE 7:**
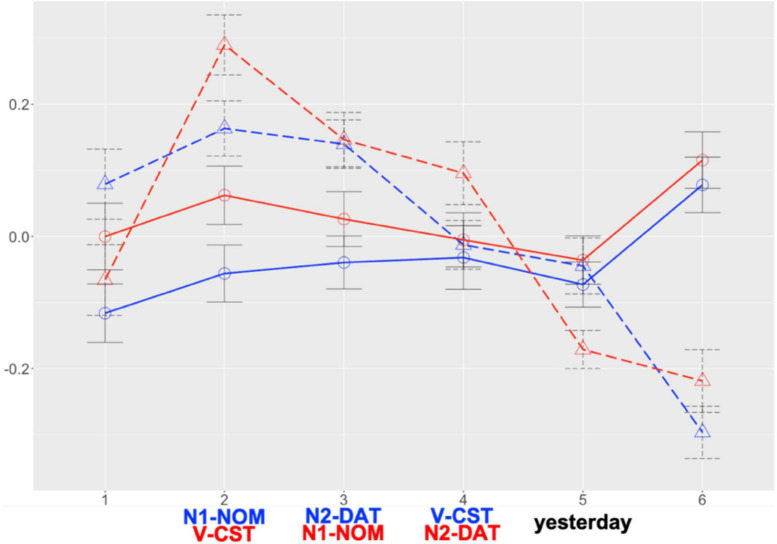
Results: Morphological causative (SPRT). *X*-axis: region; *Y*-axis: residual RT. blue: verb-final; red: verb-initial; solid line: MSK; dashed line: KHS. Error bars: 95% CI.

**FIGURE 8:**
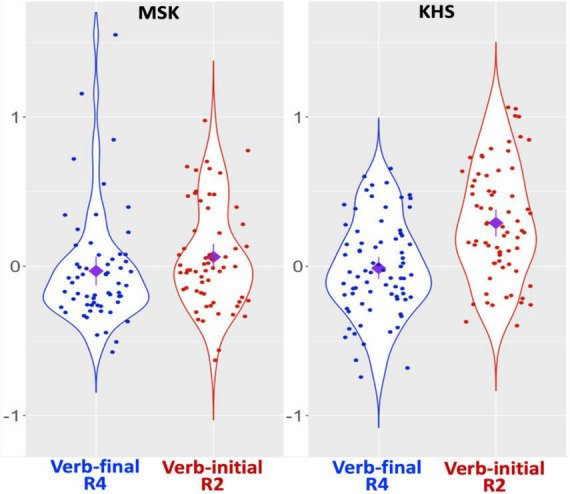
Results: Morphological causative (SPRT), verb regions only. *X*-axis: condition; *Y*-axis: residual RT. Diamond: mean; Error bars: 95% CI.

Figure 7 presents the two groups’ RT patterns (see Supplementary Appendix C, Supplementary Table A for raw RTs, log-transformed RTs, and residualised RTs per region) and Figure 8 illustrates their RT patterns focusing on verb regions. The global model (Supplementary Appendix C, Supplementary Table B) revealed main effects of *Condition* and *Group* at R2, a main effect of *Group* at R3, and an interaction effect between *Condition* and *Group* at R5. Post-hoc analyses (α = 0.025) revealed no RT difference at each region for MSK (cf. R2: numeric gap but insignificant) and a significant RT difference at R5 (β = –0.126, SE = 0.051, *t* = –2.495, *p* = 0.014) for KHS (cf. R2: numeric gap but insignificant). These indicate that, given the overall RT difference by group (R2 & R3: KHS > MSK), KHS demonstrated notable by-condition RT difference at R5 (verb-final > verb-initial). This is partially consistent with Study 1, except that by KHS at R2 and that by the two groups at R4 (Study 1: significant; Study 2: insignificant). Notably, a verb-region model (fixed effects: *Group*, *Condition*; random effect: *Word* only due to model convergence issues; α = 0.025; *R*^2^ = 0.137) revealed main effects of *Group* (β = 0.206, SE = 0.043, *t* = 4.820, *p* < 0.0005) and *Condition* (β = 0.121, SE = 0.043, *t* = 2.834, *p* = 0.005) and an interaction between the two (β = 0.208, SE = 0.086, *t* = 2.431, *p* = 0.016), with a significant by-condition difference only for KHS (β = 0.302, SE = 0.057, *t* = 5.304, *p* < 0.0005). It was further found that KHS spent less time reading R4 in the verb-final condition of Study 2 compared to Study 1 (β = –0.155, SE = 0.052, *t* = –2.954, *p* = 0.004; α = 0.0125). These findings indicate a substantial difference in the RTs that KHS allocated to the verb regions across the two conditions.

**FIGURE 9:**
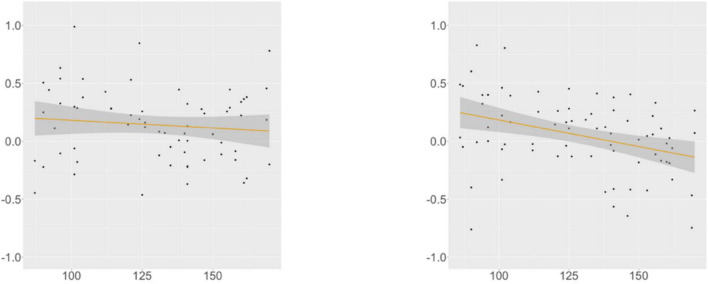
Morphological causative, KHS, R4, proficiency (*x*-axis) ∼ residual RT (*y*-axis). Gray areas: 95% CIs. Left: verb-final (*r* = −0.078, *p* = 0.507); Right: verb-initial (*r* = −0.334, *p* = 0.003).

KHS-internal models (Supplementary Appendix C, Supplementary Tables C–E) showed that each factor (*Digit*, *Flanker*, *Proficiency*) contributed to the models differently. At R4, we found a main effect of *Proficiency*; additional analyses (α = 0.0125) yielded insignificance in the verb-final condition and significance for the verb-initial condition (β = –0.005, SE = 0.002, *t* = –3.119, *p* = 0.003). This trend was supported by the correlation analysis (Figure 9): the association between the proficiency scores and the RTs was meaningful only in the verb-initial condition, indicating that KHS spent less time reading R4 in the verb-initial condition with increasing proficiency.

**FIGURE 10:**
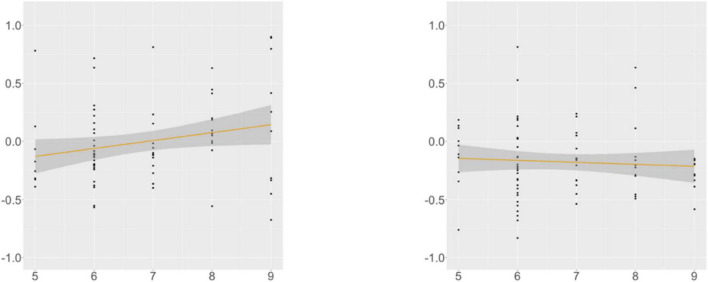
Morphological causative, KHS, R5, digit (*x*-axis) ∼ residual RT (*y*-axis). Gray areas: 95% CIs. Left: verb-final (*r* = 0.230, *p* = 0.048); Right: verb-initial (*r* = −0.070, *p* = 0.543).

**FIGURE 11:**
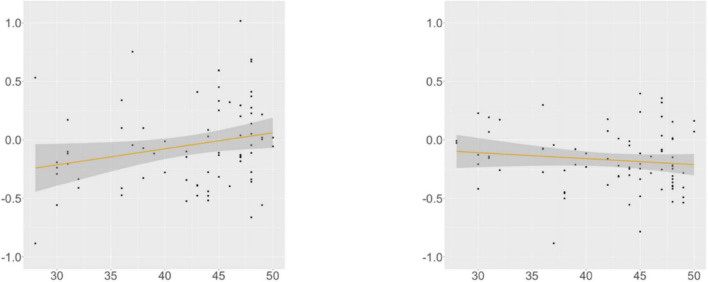
Morphological causative, KHS, R5, Flanker (*x*-axis) ∼ residual RT (*y*-axis). Gray areas: 95% CIs. Left: verb-final (*r* = 0.237, *p* = 0.039); Right: verb-initial (*r* = −0.127, *p* = 0.266).

At R5, we found a marginal interaction effect between *Condition* and *Digit* and an interaction effect between *Condition* and *Flanker*. Post-hoc analyses (α = 0.0125) yielded insignificance in both conditions, but meaningful relationships were found between the scores of the two tasks and the RTs in the verb-final condition, as shown by the correlation analysis (Figures 10, 11). These indicate that KHS spent more time reading R5 in the verb-final condition with expanding (albeit weak) IC or WM capacities.

Neither of verb-region models (fixed effects: *Condition* and one of the following factors [*Digit*, *Flanker*, *Proficiency*]; random effect: Word only due to model convergence issues; α = 0.025) revealed significant main or interaction effects (all *p*s > 0.1).

#### 3.4.2 Acceptability judgement

**FIGURE 12:**
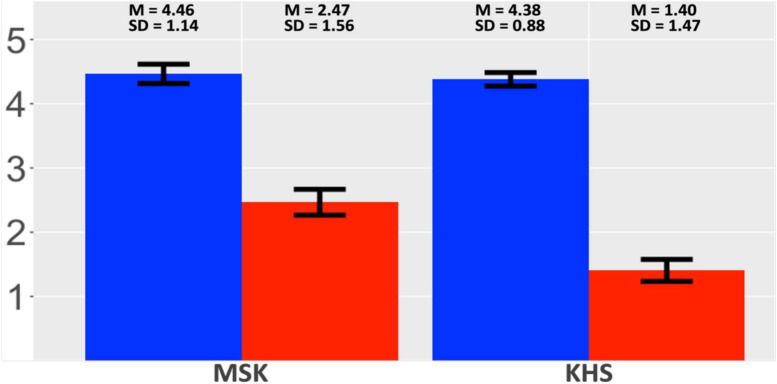
Results: Morphological causative (AJT). *X*-axis: group; *Y*-axis: acceptability. Blue: verb-final; red: verb-initial. Error bars: 95% CI.

Figure 12 presents the two groups’ acceptability-rating outcomes. Both groups rated the verb-final condition to be more acceptable than the verb-initial condition, but the by-condition gap was larger for KHS than MSK. The global model (Supplementary Appendix C, Supplementary Table F) revealed a main effect of *Condition*; additional analysis (α = 0.025) yielded insignificance for all the by-group comparisons, indicating the two groups’ uniform preference for the verb-final condition.

KHS-internal models (Supplementary Appendix C, Supplementary Tables G–I) revealed an interaction effect only in the KHS–Flanker model. This indicates that the Flanker scores modulated their ratings, driving KHS to evaluate the verb-initial condition as less acceptable with increasing IC capacities.

### 3.5 Discussion: study 2

For MSK, their RTs (particularly those involving the verb regions) and acceptability ratings for the morphological causative were almost identical to those for the suffixal passive, demonstrating no early-arriving-cue advantage and late-arriving-cue disadvantage. This corroborates the heuristic-before-algorithm strategy for sentence processing by monolingual speakers (Ferreira, 2003; Dwivedi, 2013; Kharkwal and Stromswold, 2014; Lee et al., 2023), underscoring the prominent role of heuristic parsing (prioritising canonicity/typicality involving word order and case-marking facts) over algorithmic parsing (involving interpretive procedures driven by verbal morphology) in sentence processing.

For KHS, the verb-final condition was deemed more acceptable than the verb-initial condition in AJT, and more RT was spent at R2 and R3 in both conditions compared to MSK, aligning largely with our predictions. The RT difference between the two conditions in SPRT was substantial at R5 (with the verb-final condition more time-consuming than the verb-initial condition) and at the verb regions (with R2 in the verb-initial condition more time-consuming than R4 in the verb-final condition). The insignificant by-condition difference at R2 may imply the emergence of an early-arriving-cue benefit, but this should be interpreted with caution as this insignificance seems to originate from the considerably increased RTs at this region in the verb-final condition when compared to Study 1, the reason of which is unclear. Importantly, the fact that KHS exhibited notably longer RTs when reading the verb in the verb-initial condition compared to the verb-final condition, as predicted, suggests a larger role of heuristic parsing than algorithmic parsing in sentence processing. In addition, the notable decrease in RTs at R4 in the verb-final condition of Study 2 in contrast to Study 1 suggests that, despite the cognitive demands associated with clausal integration (as indicated by the significant RT gap at R5), KHS may have leveraged the interpretive procedures involving causative morphology—presumed to be less taxing than those involving passive morphology—to some extent when coping with this region.

Pertaining to proficiency and cognitive skills, only the Flanker scores meaningfully influenced the KHS’s acceptability ratings. This differs from Study 1, and implies that construction-specific properties (e.g., alignments between thematic roles and case markers, interpretive procedures driven by verbal morphology) selectively adjust the activation of these factors and to different extents in this construction. The similar kind of trend was also found in SPRT. The proficiency and digit-span scores influenced KHS’s RT patterns in the same manner as that found in Study 1, but the Flanker scores incurred more RTs at R5 in the verb-final condition (although the effect was weak). This is inconsistent with our prediction; note that the IC effects on KHS’s RT patterns emerged at R2 in Study 1. The role of proficiency found here was the same as that in Study 1, corroborating the argument that general proficiency in a non-dominant language contributes selectively to sentence processing in that language.

The locus of this asymmetry concerning IC is ascribable to the properties of the two construction types in this study. Compared to the interpretive procedures involving the suffixal passive, those involving the morphological causative are less costly (see Sections 2.1 and 3.1). In other words, the algorithmic stream applied to each construction type differs qualitatively. This less demanding nature in the morphological causative may have allowed KHS to allocate more cognitive resources when conducting clausal integration at R5 towards a full interpretation, resulting in more RTs spent at this region (cf. Kaiser, 2014). This also aligns with why KHS spent more time at R5 in the verb-final condition proportionate to the WM scores in Study 1 (see Section 2.4).

In this respect, the null Flanker effects found at R2 in the verb-initial condition in Study 2, as well as at R2 in the verb-initial condition and at R5 in the verb-final condition in Study 1, point to the same potential mechanism underlying the non-dominant-language mind: its capacity and willingness to conduct (algorithmic) parsing induced by linguistic cues proportional to the computational complexity involving these cues. To illustrate, scrambled word order is more challenging to compute than canonical word order because scrambling is less frequently used and invites contextual/discoursal effects on interpretation. This may have overridden the presumed early-arriving-cue advantage in the verb-initial conditions of both constructions substantially. Passive morphology is more taxing than causative morphology with respect to the interpretive procedures that it drives, which may have reduced the space for the non-dominant-language mind to control information that is irrelevant to the target knowledge via IC. If this reasoning is valid, it also provides additional support for the idea that IC and WM, although interconnected, function separately and differently during sentence comprehension (Abutalebi and Green, 2007; Linck and Weiss, 2015).

## 4 Conclusion

Taken together, by examining Korean and two construction types that contrast in terms of two parsing streams, the present study has revealed the interface between a human sentence-processing architecture such as GE and various factors surrounding heritage speakers such as home-language proficiency, cognitive skills, and task types. The processor seeks efficiency when executing language activities by minimally imposing cognitive demand and processing effort, as is the case with general information processing (O’Grady, 2015; Karimi and Ferreira, 2016). In doing so, the processor strategically employs both heuristics and algorithms as a response to linguistic cues, and sometimes the heuristic parsing takes priority over the algorithmic parsing (Ferreira, 2003; Christianson, 2016). Simultaneously, diverse (non-)linguistic factors jointly adjust the way that the processor works in real time (Bice and Kroll, 2021; Torres, 2023), thereby constructing noisy representations of non-dominant-language knowledge (Futrell and Gibson, 2017; Tachihara and Goldberg, 2020). We believe that our experimental setting effectively zoomed into this aspect, which in turn advances our understanding of a learner’s mind for underrepresented languages and populations in the field.

These merits notwithstanding, we concede that the current study is constrained in its comprehensive examination of the attributes of home-language knowledge and the potential challenges associated with sentence-processing mechanisms faced by heritage speakers. Our study prompts the need for more nuanced investigations into linguistic, cognitive-psychological, and sociodemographic profiles of heritage speakers. This encompasses variations in literacy and experience of spoken/written language (cf. Karaca et al., 2024), task demands in consideration of alternative language tasks (e.g., sentence-picture matching, elicited production) (cf. Kim et al., 2018; Chondrogianni and Schwartz, 2020), and potential vulnerability of morphosyntactic knowledge itself (i.e., the extent to which they have successfully acquired the target knowledge). These areas await further exploration.

## Data availability statement

The original contributions presented in this study are included in the article/Supplementary material. Further inquiries can be directed to the corresponding author.

## Ethics statement

The studies involving humans were approved by the Office for the Protection of Research Subjects (University of Illinois at Chicago). The studies were conducted in accordance with the local legislation and institutional requirements, as well as the general practice in experimental linguistics. The participants provided their written informed consent to participate in this study.

## Author contributions

GS: Conceptualisation, Data curation, Formal analysis, Investigation, Methodology, Project administration, Resources, Software, Supervision, Validation, Visualisation, Writing – original draft, Writing – review & editing.

## Footnotes

1 See this repository for the data and code of this study.

2 Studies have shown that data collection via web-based platforms is as reliable as lab-based experimentation (Hilbig, 2016; Kim et al., 2019; Slim and Hartsuiker, 2022). Data quality was further ensured in the experimental procedure, with the individual participants’ performance supervised in real time.

3 Manipulating the existence of verbal morphology in the test stimuli (e.g., active transitive vs. suffixal passive or morphological causative) was not the focus of the current study. Our investigation proceeded with the presence of verbal morphology introducing particular interpretive procedures. Considering whether verbal morphology exists in an experimental design will open another line of inquiry into sentence processing of non-dominant languages.

4 This change may have inadvertently affected participants’ performance. The case-less noun at R1 in Study 2, in contrast to the general conversation initiator in Study 1, may provide a more conducive context for a comprehender to handle the fronted verb, potentially lowering surprisal/disequilibrium at R2 for the verb-initial condition. We concede this difference involving R1 across the two experiments, while unavoidable, could have confounded the interpretation of the results.
