# Supplementary material for: Good-enough processing, home language proficiency, cognitive skills, and task effects for Korean heritage speakers’ sentence comprehension
Source: Front Psychol. 2024 Aug 1;15:1382668. doi: 10.3389/fpsyg.2024.1382668 (PMC11324561; doi:10.3389/fpsyg.2024.1382668)
Supplement: Supplementary file 1 [file Data_Sheet_1.docx]

Supplementary Material

**Appendix A**. Test sentences: acceptability judgement, canonical word order

[Suffixal passive]

Minswu-ka Yengcay-hanthey palp-hi-ess-ta

Minswu-NOM Yengcay-DAT step.on-PSV-PST-SE

‘Minswu was step on by Yengcay.’

Mihyey-ka Cisu-hanthey ep-hi-ess-ta

Mihyey -NOM Cisu -DAT piggyback-PSV-PST-SE

‘Mihyey was piggybacked by Cisu.’

Huyswu-ka Mincay-hanthey an-ki-ess-ta

Huyswu-NOM Mincay-DAT hug-PSV-PST-SE

‘Huyswu was hugged by Mincay.’

Swuhuy-ka Hyeyli-hanthey cha-i-ess-ta

Swuhuy-NOM Hyeyli -DAT kick-PSV-PST-SE

‘Swuhuy was kicked by Hyeyli.’

Namhuy-ka Cinwu-hanthey nakk-i-ess-ta

Namhuy-NOM Chinwu-DAT hook-PSV-PST-SE

‘Namhuy was hooked by Chinwu.’

Seyhuy-ka Lia-hanthey ccik-hi-ess-ta

Seyhuy-NOM Lia-DAT spear-PSV-PST-SE

‘Seyhuy was speared by Lia.’

Minci-ka Yengmi-hanthey mwul-li-ess-ta

Minci-NOM Yengmi-DAT bite-PSV-PST-SE

‘Minci was bitten by Yengmi.’

Cinho-ka Hyeyswu-hanthey ccil-li-ess-ta

Cinho-NOM Hyeyswu-DAT poke-PSV-PST-SE

‘Cinho was poked by Hyeyswu.’

[Morphological causative]

Yengswu-ka Mincay-hanthey umsik-ul myek-y-ess-ta

Yengswu-NOM Mincay-DAT food-ACC eat-CAS-PST-SE

‘Yengswu made Mincay eat the food.’

Senhuy-ka Yenghui-hanthey sinpal-ul sin-ki-ess-ta

Senhuy-NOM Yenghui-DAT shoes-ACC wear-CAS-PST-SE

‘Senhuy made Yenghui wear the shoes.’

Mina-ka Yenci-hanthey moca-lul ssu-i-wess-ta

Mina-NOM Yenci-DAT hat-ACC wear-CAS-PST-SE

‘Mina made Yenci wear the hat.’

Sihwu-ka Chelwu-hanthey swukap-ul chay-wu-ess-ta

Sihwu-NOM Chelwu-DAT handcuffs-ACC fasten-CAS-PST-SE

‘Sihwu made Chelwu fasten the handcuffs.’

Chelswu-ka Ciyey-hanthey kapang-ul math-ki-ess-ta

Chelswu-NOM Ciyey-DAT bag-ACC take.on-CAS-PST-SE

‘Chelswu made Ciyey take on the bag.’

Sengho-ka Thayli-hanthey oythwu-lul ip-hi-ess-ta

Sengho-NOM Thayli-DAT coat-ACC wear-CAS-PST-SE

‘Seongho made Thayli wear the coat.’

Cinswu-ka Yeyci-hanthey panci-lul kki-wu-ess-ta

Cinswu-NOM Yeyci-DAT ring-ACC put.on-CAS-PST-SE

‘Cinswu made Yeyci wear the ring.’

Chelho-ka Unswu-hanthey selyu-lul nem-ki-ess-ta

Chelho-NOM Unswu -DAT document-ACC pass-CAS-PST-SE

‘Chelho made Unswu get the document.’

**Appendix B**. Model outcome by task type: Suffixal passive construction

**Table A.** SPRT: By-region reading time

|  |  | Mean (SD) | | | | | |
| --- | --- | --- | --- | --- | --- | --- | --- |
| *Raw (trimmed) in millisecond* | | R1 | R2 | R3 | R4 | R5 | R6 |
| MSK | Verb-final | 293 (121) | 306 (113) | 318 (128) | 312 (108) | 337 (129) | 353 (102) |
|  | Verb-initial | 304 (290) | 323 (133) | 330 (149) | 322 (145) | 325 (136) | 394 (144) |
| KHS | Verb-final | 830 (423) | 623 (259) | 732 (323) | 674 (317) | 585 (325) | 427 (190) |
|  | Verb-initial | 791 (450) | 804 (448) | 703 (344) | 683 (358) | 472 (218) | 461 (190) |
| *Log-transformed RT* | | R1 | R2 | R3 | R4 | R5 | R6 |
| MSK | Verb-final | 5.608 (0.377) | 5.658 (0.366) | 5.685 (0.396) | 5.683 (0.353) | 5.763 (0.331) | 5.826 (0.295) |
|  | Verb-initial | 5.649 (0.378) | 5.697 (0.413) | 5.711 (0.420) | 5.686 (0.417) | 5.707 (0.400) | 5.913 (0.362) |
| KHS | Verb-final | 6.528 (0.576) | 6.350 (0.425) | 6.490 (0.485) | 6.394 (0.511) | 6.230 (0.470) | 5.983 (0.376) |
|  | Verb-initial | 6.435 (0.656) | 6.480 (0.581) | 6.420 (0.500) | 6.380 (0.564) | 6.064 (0.425) | 6.057 (0.392) |
| *Residualised RT* | | R1 | R2 | R3 | R4 | R5 | R6 |
| MSK | Verb-final | –0.061 (0.265) | –0.072 (0.268) | –0.033 (0.258) | –0.047 (0.236) | 0.020 (0.258) | 0.095 (0.290) |
|  | Verb-initial | –0.020 (0.210) | –0.031 (0.251) | –0.019 (0.272) | –0.032 (0.270) | –0.036 (0.280) | 0.183 (0.334) |
| KHS | Verb-final | 0.038 (0.276) | 0.079 (0.249) | 0.182 (0.285) | 0.142 (0.311) | –0.005 (0.364) | –0.300 (0.317) |
|  | Verb-initial | –0.049 (0.380) | 0.224 (0.347) | 0.144 (0.274) | 0.057 (0.329) | –0.175 (0.303) | –0.233 (0.338) |

**Table B.** SPRT: Global model (α = .05)

|  |  | *β* | *SE* | *t* | *p* |
| --- | --- | --- | --- | --- | --- |
| R2 | (Intercept) | 0.056 | 0.019 | 3.018 | .003^**^ |
|  | Condition | 0.097 | 0.033 | 2.896 | .004^**^ |
|  | Group | 0.204 | 0.037 | 5.433 | < .001^***^ |
|  | Condition × Group | 0.105 | 0.067 | 1.573 | .117 |
| R3 | (Intercept) | 0.077 | 0.019 | 4.052 | < .001^***^ |
|  | Condition | –0.015 | 0.037 | –0.392 | .700 |
|  | Group | 0.189 | 0.033 | 5.688 | < .001^***^ |
|  | Condition × Group | –0.052 | 0.065 | –0.800 | .425 |
| R4 | (Intercept) | 0.034 | 0.018 | 1.935 | .054 |
|  | Condition | –0.039 | 0.035 | –1.092 | .276 |
|  | Group | 0.138 | 0.035 | 3.885 | < .001^***^ |
|  | Condition × Group | –0.100 | 0.071 | –1.408 | .160 |
| R5 | (Intercept) | –0.054 | 0.018 | –2.963 | .003^**^ |
|  | Condition | –0.119 | 0.037 | –3.239 | .001^**^ |
|  | Group | –0.083 | 0.037 | –2.266 | .024^*^ |
|  | Condition × Group | –0.114 | 0.074 | –1.550 | .122 |

**Table C.** SPRT: KHS model with Digit (α = .025)

|  |  | *β* | *SE* | *t* | *p* |
| --- | --- | --- | --- | --- | --- |
| R2 | (Intercept) | 0.154 | 0.160 | 0.962 | .342 |
|  | Condition | 0.345 | 0.257 | 1.341 | .183 |
|  | Digit | –0.0003 | 0.023 | –0.016 | .987 |
|  | Condition * Digit | –0.029 | 0.037 | –0.786 | .434 |
| R3 | (Intercept) | 0.246 | 0.126 | 1.948 | .059 |
|  | Condition | 0.205 | 0.250 | 0.821 | .413 |
|  | Digit | –0.012 | 0.018 | –0.680 | .501 |
|  | Condition * Digit | –0.036 | 0.036 | –1.002 | .319 |
| R4 | (Intercept) | 0.232 | 0.149 | 1.555 | .122 |
|  | Condition | 0.232 | 0.300 | 0.773 | .441 |
|  | Digit | –0.020 | 0.022 | –0.937 | .350 |
|  | Condition * Digit | –0.047 | 0.043 | –1.090 | .278 |
| R5 | (Intercept) | –0.260 | 0.148 | –1.752 | .081 |
|  | Condition | 0.409 | 0.297 | 1.377 | .171 |
|  | Digit | 0.024 | 0.021 | 1.134 | .258 |
|  | Condition * Digit | –0.085 | 0.043 | –1.976 | .050 |

**Table D.** SPRT: KHS model with Flanker (α = .025)

|  |  | *β* | *SE* | *t* | *p* |
| --- | --- | --- | --- | --- | --- |
| R2 | (Intercept) | 0.311 | 0.197 | 1.574 | .123 |
|  | Condition | –0.597 | 0.322 | –1.854 | .066 |
|  | Flanker | –0.004 | 0.004 | –0.817 | .419 |
|  | Condition * Flanker | 0.017 | 0.007 | 2.333 | .021* |
| R3 | (Intercept) | 0.163 | 0.158 | 1.031 | .309 |
|  | Condition | –0.059 | 0.313 | –0.191 | .849 |
|  | Flanker | < 0.001 | 0.004 | 0.000 | 1.000 |
|  | Condition * Flanker | < 0.001 | 0.007 | 0.069 | .945 |
| R4 | (Intercept) | 0.286 | 0.182 | 1.565 | .120 |
|  | Condition | 0.272 | 0.367 | 0.742 | .459 |
|  | Flanker | –0.004 | 0.004 | –1.049 | .296 |
|  | Condition * Flanker | –0.008 | 0.008 | –0.991 | .323 |
| R5 | (Intercept) | –0.034 | 0.191 | –0.179 | .858 |
|  | Condition | –0.491 | 0.383 | –1.280 | .203 |
|  | Flanker | –0.001 | 0.004 | –0.307 | .759 |
|  | Condition * Flanker | 0.007 | 0.009 | 0.843 | .401 |

**Table E.** SPRT: KHS model with Proficiency (α = .025)

|  |  | *β* | *SE* | *t* | *p* |
| --- | --- | --- | --- | --- | --- |
| R2 | (Intercept) | 0.316 | 0.149 | 2.120 | .040 |
|  | Condition | 0.445 | 0.244 | 1.826 | .071 |
|  | Proficiency | –0.001 | 0.001 | –1.114 | .272 |
|  | Condition * Proficiency | –0.002 | 0.001 | –1.241 | .217 |
| R3 | (Intercept) | 0.312 | 0.113 | 2.756 | .007* |
|  | Condition | 0.398 | 0.227 | 1.755 | .081 |
|  | Proficiency | –0.001 | 0.001 | –1.364 | .175 |
|  | Condition * Proficiency | –0.003 | 0.002 | –1.985 | .049 |
| R4 | (Intercept) | 0.485 | 0.132 | 3.680 | < .0005*** |
|  | Condition | 0.328 | 0.264 | 1.239 | .217 |
|  | Proficiency | –0.003 | 0.001 | –3.011 | .003** |
|  | Condition * Proficiency | –0.003 | 0.002 | –1.615 | .108 |
| R5 | (Intercept) | –0.114 | 0.136 | –0.837 | .404 |
|  | Condition | –0.161 | 0.273 | –0.590 | .556 |
|  | Proficiency | < 0.0001 | 0.001 | 0.160 | .873 |
|  | Condition * Proficiency | < –0.0001 | 0.002 | –0.034 | .973 |

**Table F.** AJT: Global model (*α* = .05)

|  | *β* | *SE* | *t* | *p* |
| --- | --- | --- | --- | --- |
| (Intercept) | < .001 | 0.030 | –0.001 | .999 |
| Condition | –1.412 | 0.060 | –23.343 | < .001^***^ |
| Group | < .001 | 0.060 | 0.010 | .992 |
| Condition × Group | 0.296 | 0.121 | 2.440 | .015^*^ |

**Table G.** AJT: KHS model with Digit (*α* = .025)

|  | *β* | *SE* | *t* | *p* |
| --- | --- | --- | --- | --- |
| (Intercept) | 0.018 | 0.254 | 0.072 | .943 |
| Condition | –0.074 | 0.509 | –0.146 | .884 |
| Digit | –0.003 | 0.037 | –0.080 | .937 |
| Condition * Digit | –0.178 | 0.074 | –2.399 | .018^*^ |

**Table H.** AJT: KHS model with Flanker (*α* = .025)

|  | *β* | *SE* | *t* | *p* |
| --- | --- | --- | --- | --- |
| (Intercept) | –0.071 | 0.336 | –0.213 | .832 |
| Condition | 0.303 | 0.671 | 0.452 | .652 |
| Flanker | 0.002 | 0.008 | 0.219 | .827 |
| Condition * Flanker | –0.037 | 0.016 | –2.371 | .019^*^ |

**Table I.** AJT: KHS model with Proficiency (*α* = .025)

|  | *β* | *SE* | *t* | *p* |
| --- | --- | --- | --- | --- |
| (Intercept) | –0.025 | 0.232 | –0.107 | .915 |
| Condition | –0.221 | 0.463 | –0.478 | .634 |
| Proficiency | < .001 | 0.002 | 0.113 | .910 |
| Condition * Proficiency | –0.008 | 0.003 | –2.319 | .022^*^ |

**Appendix C**. Model outcome by task type: Morphological causative construction

**Table A.** SPRT: By-region reading time

|  |  | Mean (SD) | | | | | |
| --- | --- | --- | --- | --- | --- | --- | --- |
| *Raw (trimmed) in millisecond* | | R1 | R2 | R3 | R4 | R5 | R6 |
| MSK | Verb-final | 311 (163) | 334 (202) | 325 (138) | 349 (260) | 308 (113) | 352 (124) |
|  | Verb-initial | 379 (295) | 373 (197) | 358 (206) | 351 (210) | 323 (144) | 367 (126) |
| KHS | Verb-final | 977 (473) | 743 (357) | 764 (357) | 613 (296) | 561 (286) | 446 (192) |
|  | Verb-initial | 935 (513) | 841 (441) | 718 (322) | 778 (455) | 468 (164) | 508 (317) |
| *Log-transformed RT* | | R1 | R2 | R3 | R4 | R5 | R6 |
| MSK | Verb-final | 5.629 (0.453) | 5.680 (0.493) | 5.698 (0.419) | 5.704 (0.500) | 5.661 (0.380) | 5.814 (0.314) |
|  | Verb-initial | 5.747 (0.573) | 5.799 (0.490) | 5.763 (0.468) | 5.733 (0.481) | 5.692 (0.410) | 5.849 (0.340) |
| KHS | Verb-final | 6.733 (0.604) | 6.491 (0.507) | 6.524 (0.500) | 6.312 (0.463) | 6.219 (0.470) | 6.028 (0.374) |
|  | Verb-initial | 6.634 (0.714) | 6.589 (0.566) | 6.463 (0.507) | 6.477 (0.626) | 6.087 (0.363) | 6.101 (0.477) |
| *Residualised RT* | | R1 | R2 | R3 | R4 | R5 | R6 |
| MSK | Verb-final | –0.116 (0.346) | –0.056 (0.337) | –0.039 (0.312) | –0.032 (0.376) | –0.073 (0.267) | 0.078 (0.328) |
|  | Verb-initial | 0.000 (0.397) | 0.062 (0.348) | 0.026 (0.328) | –0.005 (0.324) | –0.036 (0.285) | 0.115 (0.334) |
| KHS | Verb-final | 0.079 (0.425) | 0.163 (0.364) | 0.140 (0.311) | –0.013 (0.319) | –0.045 (0.369) | –0.297 (0.342) |
|  | Verb-initial | –0.066 (0.425) | 0.290 (0.377) | 0.147 (0.356) | 0.096 (0.413) | –0.171 (0.255) | –0.219 (0.416) |

**Table B.** SPRT: Global model (α = .05)

|  |  | *β* | *SE* | *t* | *p* |
| --- | --- | --- | --- | --- | --- |
| R2 | (Intercept) | 0.122 | 0.024 | 5.069 | < .001^***^ |
|  | Condition | 0.124 | 0.045 | 2.761 | .018^*^ |
|  | Group | 0.224 | 0.046 | 4.843 | < .001^***^ |
|  | Condition × Group | 0.008 | 0.085 | 0.098 | .922 |
| R3 | (Intercept) | 0.074 | 0.020 | 3.613 | .004^**^ |
|  | Condition | 0.032 | 0.041 | 0.773 | .457 |
|  | Group | 0.149 | 0.040 | 3.752 | < .001^***^ |
|  | Condition × Group | –0.059 | 0.079 | –0.746 | .456 |
| R4 | (Intercept) | 0.018 | 0.026 | 0.688 | .501 |
|  | Condition | 0.077 | 0.052 | 1.491 | .155 |
|  | Group | 0.060 | 0.043 | 1.393 | .165 |
|  | Condition × Group | 0.081 | 0.086 | 0.938 | .349 |
| R5 | (Intercept) | –0.084 | 0.018 | –4.724 | < .001^***^ |
|  | Condition | –0.054 | 0.036 | –1.515 | .131 |
|  | Group | –0.054 | 0.036 | –1.502 | .134 |
|  | Condition × Group | –0.163 | 0.072 | –2.267 | .024^*^ |

**Table C.** SPRT: KHS model with Digit (α = .025)

|  |  | *β* | *SE* | *t* | *p* |
| --- | --- | --- | --- | --- | --- |
| R2 | (Intercept) | 0.109 | 0.191 | 0.568 | .573 |
|  | Condition | 0.256 | 0.319 | 0.802 | .424 |
|  | Digit | 0.017 | 0.028 | 0.627 | .535 |
|  | Condition * Digit | –0.019 | 0.046 | –0.423 | .674 |
| R3 | (Intercept) | –0.111 | 0.152 | –0.731 | .466 |
|  | Condition | –0.070 | 0.305 | –0.230 | .818 |
|  | Digit | 0.038 | 0.022 | 1.697 | .092 |
|  | Condition * Digit | 0.011 | 0.044 | 0.256 | .798 |
| R4 | (Intercept) | 0.163 | 0.160 | 1.017 | .311 |
|  | Condition | –0.090 | 0.321 | –0.282 | .778 |
|  | Digit | –0.017 | 0.023 | –0.754 | .452 |
|  | Condition * Digit | 0.030 | 0.046 | 0.655 | .513 |
| R5 | (Intercept) | –0.233 | 0.137 | –1.692 | .093 |
|  | Condition | 0.479 | 0.275 | 1.742 | .083 |
|  | Digit | 0.018 | 0.020 | 0.914 | .362 |
|  | Condition * Digit | –0.090 | 0.040 | –2.238 | .027 |

**Table D.** SPRT: KHS model with Flanker (α = .025)

|  |  | *β* | *SE* | *t* | *p* |
| --- | --- | --- | --- | --- | --- |
| R2 | (Intercept) | 0.241 | 0.234 | 1.031 | .309 |
|  | Condition | 0.771 | 0.386 | 1.995 | .049 |
|  | Flanker | < –0.001 | 0.005 | –0.065 | .949 |
|  | Condition * Flanker | –0.015 | 0.009 | –1.693 | 094 |
| R3 | (Intercept) | 0.048 | 0.182 | 0.266 | .790 |
|  | Condition | 0.451 | 0.363 | 1.242 | .216 |
|  | Flanker | 0.002 | 0.004 | 0.525 | .600 |
|  | Condition * Flanker | –0.010 | 0.008 | –1.239 | .217 |
| R4 | (Intercept) | 0.433 | 0.193 | 2.239 | .027 |
|  | Condition | 0.567 | 0.387 | 1.466 | .145 |
|  | Flanker | –0.009 | 0.004 | –2.041 | .043 |
|  | Condition * Flanker | –0.011 | 0.009 | –1.194 | .234 |
| R5 | (Intercept) | –0.285 | 0.167 | –1.702 | .091 |
|  | Condition | 0.670 | 0.335 | 1.999 | .047 |
|  | Flanker | 0.004 | 0.004 | 1.062 | .290 |
|  | Condition * Flanker | –0.019 | 0.008 | –2.401 | .017* |

**Table E.** SPRT: KHS model with Proficiency (α = .025)

|  |  | *β* | *SE* | *t* | *p* |
| --- | --- | --- | --- | --- | --- |
| R2 | (Intercept) | 0.504 | 0.170 | 2.965 | .005^*^ |
|  | Condition | 0.103 | 0.292 | 0.354 | .724 |
|  | Proficiency | –0.002 | 0.001 | –1.674 | .102 |
|  | Condition * Proficiency | < 0.001 | 0.002 | 0.083 | .934 |
| R3 | (Intercept) | 0.262 | 0.139 | 1.880 | .062 |
|  | Condition | –0.182 | 0.278 | –0.653 | .515 |
|  | Proficiency | < –0.001 | 0.001 | –-0.871 | .385 |
|  | Condition * Proficiency | 0.001 | 0.002 | 0.682 | .496 |
| R4 | (Intercept) | 0.442 | 0.142 | 3.109 | .002^**^ |
|  | Condition | 0.626 | 0.284 | 2.203 | .029 |
|  | Proficiency | –0.003 | 0.001 | –2.882 | .004^**^ |
|  | Condition * Proficiency | –0.004 | 0.002 | –1.862 | .065 |
| R5 | (Intercept) | –0.131 | 0.127 | –1.030 | .305 |
|  | Condition | –0.406 | 0.254 | –1.601 | .111 |
|  | Proficiency | < 0.001 | < 0.001 | 0.174 | .862 |
|  | Condition * Proficiency | 0.002 | 0.002 | 1.125 | .262 |

**Table F.** AJT: Global model (*α* = .05)

|  | *β* | *SE* | *t* | *p* |
| --- | --- | --- | --- | --- |
| (Intercept) | < .001 | 0.034 | 0.014 | .989 |
| Condition | –1.413 | 0.067 | –20.789 | < .001^***^ |
| Group | < .001 | 0.060 | 0.053 | .958 |
| Condition × Group | –0.056 | 0.121 | -0.459 | .647 |

**Table G.** AJT: KHS model with Digit (*α* = .025)

|  | *β* | *SE* | *t* | *p* |
| --- | --- | --- | --- | --- |
| (Intercept) | –0.015 | 0.202 | –0.073 | .942 |
| Condition | –1.035 | 0.403 | –2.565 | .011^*^ |
| Digit | 0.002 | 0.030 | 0.079 | .937 |
| Condition * Digit | –0.059 | 0.059 | –0.998 | .320 |

**Table H.** AJT: KHS model with Flanker (*α* = .025)

|  | *β* | *SE* | *t* | *p* |
| --- | --- | --- | --- | --- |
| (Intercept) | –0.010 | 0.267 | –0.040 | .968 |
| Condition | 0.147 | 0.534 | 0.275 | .784 |
| Flanker | < .001 | 0.006 | 0.046 | .963 |
| Condition * Flanker | –0.037 | 0.012 | –2.993 | .003^**^ |

**Table I.** AJT: KHS model with Proficiency (*α* = .025)

|  | *β* | *SE* | *t* | *p* |
| --- | --- | --- | --- | --- |
| (Intercept) | –0.047 | 0.198 | –0.239 | .811 |
| Condition | –1.077 | 0.396 | –2.716 | .007^*^ |
| Proficiency | < .001 | 0.001 | 0.252 | .801 |
| Condition * Proficiency | –0.002 | 0.003 | –0.911 | .364 |
